# Calcification of Human Saphenous Vein Associated with Endothelial Dysfunction: A Pilot Histopathophysiological and Demographical Study

**DOI:** 10.3389/fsurg.2017.00006

**Published:** 2017-02-09

**Authors:** Sydney L. Pedigo, Christy M. Guth, Kyle M. Hocking, Alex Banathy, Fan Dong Li, Joyce Cheung-Flynn, Colleen M. Brophy, Raul J. Guzman, Padmini Komalavilas

**Affiliations:** ^1^Department of Surgery, Vanderbilt University Medical Center, Nashville, TN, USA; ^2^Veterans Affairs Tennessee Valley Healthcare System, Nashville, TN, USA; ^3^Beth Israel Deaconess Medical Center, Boston, MA, USA

**Keywords:** calcification, demographics, intimal thickness, endothelial dysfunction, endothelial-dependent relaxation, vein graft failure, human saphenous vein

## Abstract

While the pathophysiology and clinical significance of arterial calcifications have been studied extensively, minimal focus has been placed on *venous* calcification deposition. In this study, we evaluated the association between calcium deposition in human saphenous vein (HSV), endothelial function, and patient demographic risk factors. Fifty-four HSV segments were collected at the time of coronary artery bypass graft (CABG) surgery. The presence or absence of calcium deposits was visualized using the Von Kossa staining method. Endothelial function was determined by measuring muscle tissue contraction with phenylephrine and relaxation with carbachol in a muscle bath. Additional segments of vein underwent histologic evaluation for preexisting intimal thickness and extracellular matrix (ECM) deposition. Patient demographics data were obtained through our institution’s electronic medical record, with patient consent. Calcium was present in 16 of 54 samples (29.6%). Veins with calcium deposits had significantly greater intimal-to-medial thickness ratios (*p* = 0.0058) and increased extracellular collagen deposition (*p* = 0.0077). Endothelial relaxation was significantly compromised in calcified veins vs. those without calcium (*p* = 0.0011). Significant patient risk factors included age (*p* = 0.001) and preoperative serum creatinine (*p* = 0.017). Calcified veins can be characterized as having endothelial dysfunction with increased basal intimal thickness and increased ECM deposition. Patient risk factors for calcium deposits in veins were similar to those for arteries, namely, advanced age and kidney disease. Further studies are needed to determine the effect of preexisting vein calcification on short- and long-term graft patency.

## Introduction

Saphenous vein is the most commonly used bypass conduit for coronary artery and peripheral arterial bypass surgery. However, recent large prospective studies have indicated that graft failure rates remain high [39% of lower extremity bypass grafts and 45% of coronary artery bypass grafts (CABGs) will fail within 12–18 months, Project of *Ex vivo* Vein Graft Engineering *via* Transfection (PREVENT) III and PREVENT IV trials, respectively] (1, 2). The leading cause of graft failure is intimal hyperplasia (IH), a complex process involving the migration, proliferation, and phenotypic modulation of vascular smooth muscle cells (VSMCs) (3).

Preexisting disease in the saphenous vein has been implicated as a contributing factor to the development of IH and graft failure. The most frequently encountered lesion found on pre-bypass vein histology is intimal thickening (4). Our lab has previously published an association between preexisting intimal thickness and endothelial dysfunction (5). Marin et al. reported more frequent peripheral bypass graft failure with use of thicker-walled saphenous vein (6). Additional characteristics in morphology may contribute to the baseline quality of a saphenous vein graft.

Studies conducted to determine the quality of *arterial* conduits for CABG (most commonly the internal mammary and radial arteries) have evaluated vessel wall thickness and vessel wall calcification (7–9). In general, the pathophysiology and clinical relevance of arterial calcification is much better established than for venous calcification. Arterial calcification is broadly categorized by its vascular layer of involvement. Medial calcification, or Monckeberg sclerosis, is generally associated with metabolic derangement, such as in type 2 diabetes mellitus, end-stage renal disease, and hyperphosphatemia, and results in reduced arterial compliance and elasticity (10–12). The subsequent widening of pulse pressure is an independent predictor of hypertension, stroke, and all-cause mortality. In contrast, intimal calcification, a component of atherosclerosis, is more often associated with inflammatory states, such as in hyperlipidemia, hypertension, and smoking. These calcified plaques contribute to the narrowing of the arterial lumen and increase the risk of ischemic events. Coronary artery calcification of either type is an independent predictor of cardiovascular mortality and is used as a surrogate end point for clinical trials to determine the efficacy of medications and lifestyle modification (13). Since saphenous vein is the most widely used conduit for CABG, a study of vein calcification will help to understand how it affects the quality of the vein as a conduit and whether it contributes to the development of IH and subsequent graft failure.

In this study, we evaluated saphenous vein segments, obtained at the time of CABG surgery, for preexisting calcification. The presence or absence of calcium deposits was then correlated with other histologic markers of venous pathology (intimal thickness and ECM deposition), physiologic function, and patient demographics. We hypothesized that the presence of calcium deposits would be associated with concurrent venous pathology and physiologic dysfunction. In addition, we anticipated that known patient risk factors for arterial calcification would also predict the presence of venous calcification.

## Materials and Methods

### Procurement of Human Saphenous Veins (HSVs)

Human saphenous vein samples were collected at the time of harvest for CABG surgery. This study was carried out in accordance with the recommendations of the Institutional Review Boards of Vanderbilt University Medical Center and the VA Tennessee Valley Healthcare System in Nashville, TN, USA with written informed consent from all subjects. All subjects gave written informed consent in accordance with the Declaration of Helsinki. The protocol was approved by the Institutional Review Boards of Vanderbilt University Medical Center and the VA Tennessee Valley Healthcare System in Nashville, TN, USA. Segments of vein were harvested from 54 patients by either open or endoscopic technique, according to surgeon preference. Samples were immediately stored in sterile, heparinized (10 U/mL) PlasmaLyte (Baxter Healthcare Corporation, Deerfield, IL, USA) solution (140 mEq sodium, 5 mEq potassium, 3 mEq magnesium, 98 mEq chloride, 27 mEq acetate, and 23 mEq gluconate) without further manipulation. Tissue with obvious forceps crush injury and areas containing a tributary orifice were discarded. The remaining tissue was then cut into sequential, 2 mm rings, fixed with 10% buffered formalin, and sent to the Translational Pathology Shared Resource at Vanderbilt University Medical Center for histologic preparation of 5 µm sequential sections embedded in paraffin. Additional fresh tissue rings were cut and suspended in a muscle bath to evaluate physiologic properties.

### Evaluation of Calcium Deposits

Vein ring sections were stained using the Von Kossa method to observe calcification. Paraffin was melted and removed from each slide. Slides were then incubated in 5% silver nitrate solution for 2 h under a 50 W light (control slide was incubated in water). Slides were then washed with 5% sodium thiosulfate, counterstained with hematoxylin, dehydrated, and mounted. Tissue images were obtained and digitalized using the Zeiss Axiovert 200 M microscope at 20× magnification. Veins were then grouped as having no calcium deposits (non-calcified) or having some amount of calcium deposits present (calcified).

### Intimal Thickness Measurement

Verhoeff-Van Gieson (VVG) stain was used to visualize the vein intima and media. A total of eight intimal width measurements were made from each image, two from each quadrant at 20×. Vein intimal thickness for each sample was defined as the mean of these measurements. The medial thickness was obtained in a similar fashion at 5× magnification, and the intimal/medial ratio (IMR) was calculated.

### Extracellular Matrix (ECM) Deposition Analysis

Extracellular matrix deposition was examined using a Movat pentachrome stain, which gives collagen a yellow-brown hue. The collagen amount was quantified using Photoshop C5’s pixel count feature at a fuzziness level of 50%. The pixel count was standardized as a ratio to the overall image pixel number. At least four pictures from each vein sample were analyzed and the ratios averaged.

### Physiologic Measurements of HSVs

Details regarding the use of muscle baths to determine vascular physiologic function have been previously described (5). Briefly, 1 mm rings from the tissue were cut, weighed, and measured lengthwise using calipers. Rings were suspended in an organ bath containing a bicarbonate buffer (120 mM NaCl, 4.7 mM KCl, 1.0 mM MgSO_4_, 1.0 mM NaH_2_PO_4_, 10 mM glucose, 1.5 mM CaCl_2_, and 25 mM Na_2_HCO_3_, pH 7.4), equilibrated with 95% oxygen and 5% carbon dioxide at 37°C. Each ring was progressively stretched to its optimal resting tension (approximately 1 g) that would produce a maximal response to contractile agonists, then maintained at the resting tension and equilibrated for a minimum of 2 h. Force measurements were obtained using a Radnoti Glass Technology (Monrovia, CA, USA) force transducer (159901A) interfaced with a Powerlab data acquisition system and Chart software (ADInstruments, Colorado Springs, CO, USA). The rings were contracted with 110mM potassium chloride (KCl) to determine functional viability. Samples that failed to contract with KCl were considered non-functional and removed from further testing. Functional rings were equilibrated in the bicarbonate buffer for 30 min and were then contracted with phenylephrine (10^−6^M), and the endothelium-dependent relaxation was measured with carbachol (5 × 10^−7^M), a cholinergic agonist that causes tissue relaxation in the presence of intact endothelium. The force generated was measured and converted to stress {(10^5^ N/m^2^) = force (g) × 0.0987/area, where area is equal to the wet weight [(mg)/length (mm at maximal length)] divided by 1.055} using each specimen’s weight and length. The percentage of endothelial-dependent relaxation was calculated as the change in stress compared to the maximal tension induced by phenylephrine as previously described (14).

### Patient Demographic Collection

Patient demographics and lab values were obtained using institutional electronic medical records with patient consent. The data collected included demographic characteristics, laboratory results, comorbidities, and medications listed at the time of surgery. Patients were grouped by the presence or absence of preexisting saphenous vein calcification.

### Statistical Analysis

Bivariate analysis of all variables was conducted to screen for those factors associated with calcification (*via* Pearson chi-squared test). Factors that trended with calcification were included in logistic multivariate stepwise regression models. Variables significant upon multivariate analysis were deemed independent predictors of the end points, at a level of statistical significance of *p* < 0.05. As all variables included in multivariate analyses had a variance inflation factor of <1.8, collinearity was not statistically considered. Measures of central tendency were reported as mean ± SD. Statistical analysis was performed with JMP Pro 11 (Cary, NC, USA) and GraphPad Prism (La Jolla, CA, USA).

## Results

A total of 54 vein segments were collected and analyzed for the presence of calcification. Forty of these samples underwent additional histologic analysis to evaluate preexisting intimal thickness and ECM deposition. The remaining 14 segments were excluded due to improper histologic processing. All 54 vein segments underwent physiologic testing on muscle baths. Of these, 39 were viable based on the KCl contraction challenge. Finally, we obtained baseline demographic data on 53 patients.

### Presence of Calcium Associated with Preexisting Intimal Thickness

**Figure 1:**
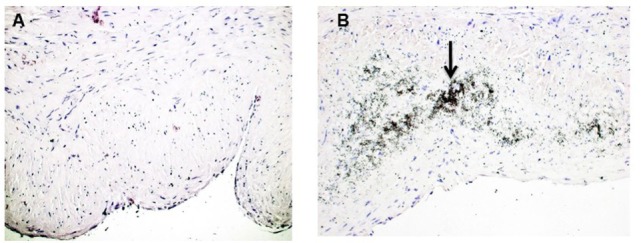
**Calcification in human saphenous veins (HSVs)**. HSV rings were stained with the Von Kossa stain to detect calcium deposits. **(A)** Von Kossa stained veins without calcification. **(B)** Veins that stained positive for calcification contained black, speckled area (black arrow, 20×) that indicated calcium deposits.

**Figure 2:**
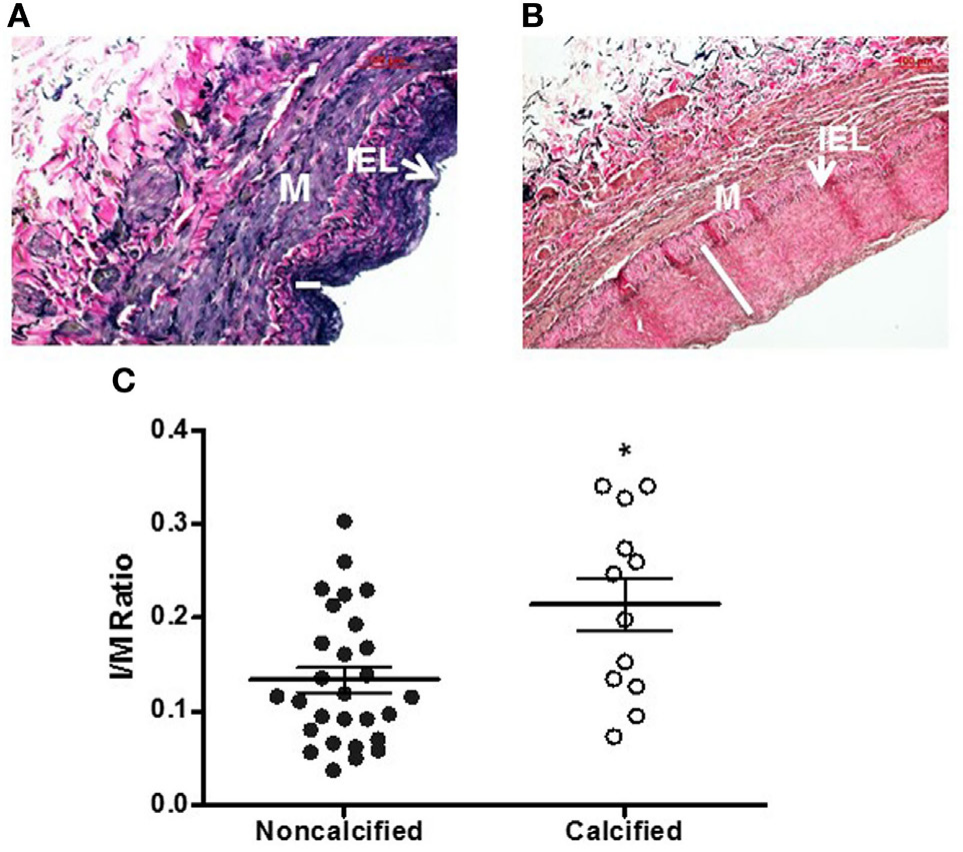
**Intimal thickening in human saphenous veins (HSVs) [Verhoeff-Van Gieson (VVG) stain]**. HSVs were fixed, sectioned, and stained with VVG and imaged with light microscopy. Representative images of non-calcified vein with thin intima [**(A)**, 20×] and calcified vein with thick intima [**(B)**, 20×]. White line indicates the thickness of intima. IEL, internal elastic lamina, M, media. **(C)** The intima to media (I/M) ratio of calcified veins compared to non-calcified veins was significantly higher (*p = 0.0058, *n* = 12–28).

Calcium deposits were present in 16 of 54 vein samples (29.6%) (Figure 1). Calcified veins had greater preexisting intimal thickness, expressed IMR, compared to non-calcified veins (0.214 ± 0.028 vs. 0.134 ± 0.013; *p* = 0.006) (Figure 2).

### Calcification Associated with Additional Markers of Vascular Pathology

**Figure 3:**
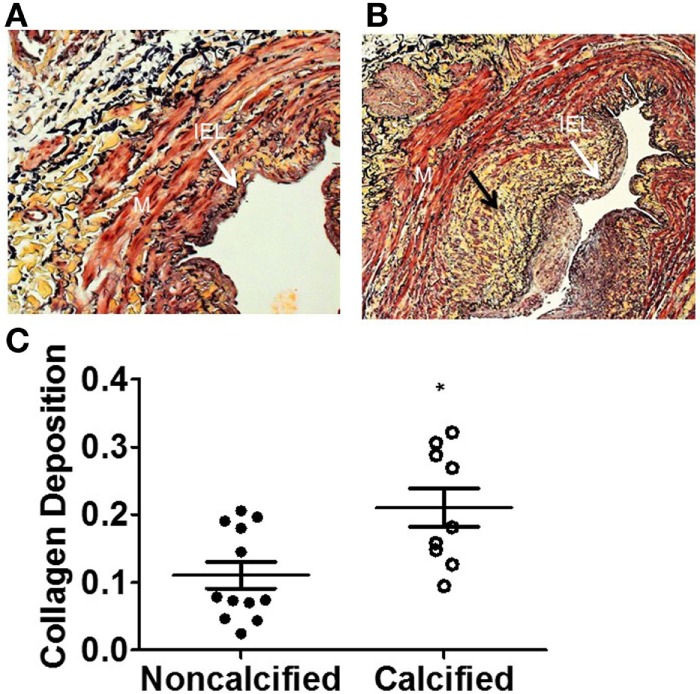
**Increased extracellular matrix (ECM) deposition in human saphenous veins with calcification**. The amount of ECM deposition was evaluated using the Movat pentachrome stain. The yellow areas (black arrow) represent collagen in non-calcified vein **(A)** and calcified vein **(B)**. **(C)** Quantitation of collagen. The calcified veins had a significantly higher amount of ECM deposition compared to non-calcified veins (*p* = 0.0182, *n* = 10–12).

We examined whether calcified veins would exhibit other histologic markers of pathology. ECM collagen deposition is a component of the IH response (15). Calcified veins revealed more ECM collagen deposition, expressed as a pixel ratio, overall (0.210 ± 0.029 vs. 0.111 ± 0.020; *p* = 0.0077). It was notable that collagen deposition appeared to localize with calcium with areas of increased calcium and collagen density within the tissue ring (Figure 3).

### Venous Calcification Inversely Correlated with Endothelial-Dependent Relaxation

**Figure 4:**
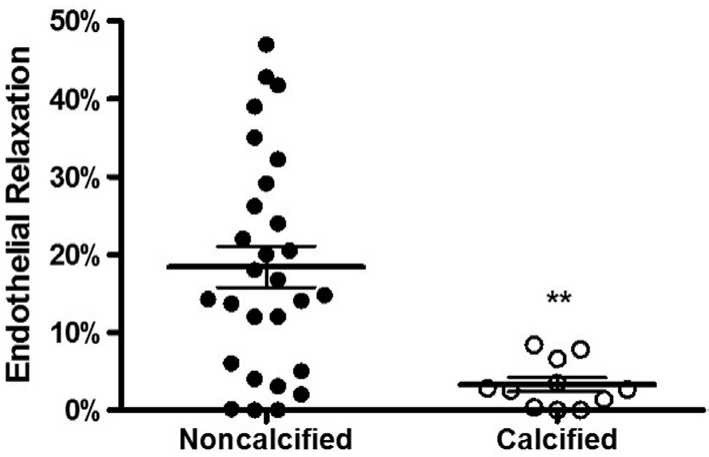
**Calcification and endothelial relaxation of human saphenous vein**. The vein segments were equilibrated in a muscle bath and contracted with phenylephrine (10^−6^ M) and relaxed with carbachol (10^−7^ M) to measure the endothelial relaxation. The endothelial relaxation of veins with calcification was significantly lower compared to non-calcified veins (*p* = 0.0011, *n* = 12–28).

We then examined whether calcification affected the endothelial function of a vein. One reliable method of assessing vessel function is endothelial relaxation. Vein rings were contracted with phenylephrine (10^−6^ M) and treated with carbachol (5 × 10^−7^ M). In the presence of an intact, functioning endothelium, carbachol induces relaxation of the vessel. Thirty-nine vein samples were determined to be viable, and the percentage of carbachol-induced relaxation was calculated. The endothelial relaxation of calcified veins was lower in comparison to non-calcified veins (3.3 ± 0.9 vs. 18.4 ± 3.0%, *p* = 0.0011, Figure 4).

### Patient Age and Kidney Function Predict Venous Calcifications

**Figure 5:**
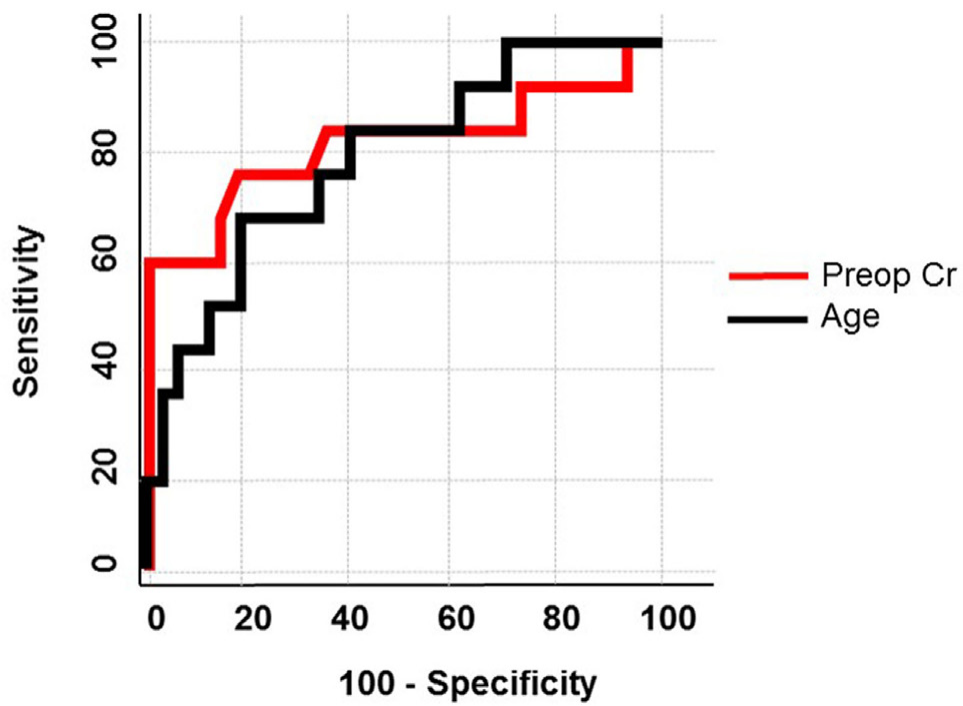
**Receiver operator curve using age and preoperative creatinine**. In a multivariate logistic regression, age and preoperative creatinine were used to predict calcification of human saphenous veins resulting in an area under the curve of 0.78 and 0.81, respectively.

Patient data were collected and paired with each vein sample. Patient groups were defined as those without evidence of venous calcium (non-calcified) and those with any amount of calcium (calcified). Using the Pearson chi-squared test to determine which factors were associated with calcification the following had a strong association with calcification: age, ever smoker, chronic kidney disease (CKD), preoperative serum creatinine (SCr), and glomerular filtration rate. After running a stepwise regression fit using minimum Bayesian information criterion as a stopping rule, age and preoperative SCr were entered into the model. A nominal logistic regression of these two variables yielded significance for both variables in a multivariate analysis. Preoperative SCr had an area under the curve of 0.81, *p* = 0.001, and a unit odds ratio of 405.7, age had an area under the curve of 0.78, *p* = 0.003, and a unit odds ratio of 1.12 (Figure 5; Table 1).

**Table 1:** **Demographic data on patients with calcium deposits or no calcium deposits within a saphenous vein sample**.

Patient characteristics	Non-calcified vein (*n* = 33)	Calcified vein (*n* = 12)	*p*
Age	60.5 ± 1.8	72.3 ± 2.9	**0.001**
Male sex	85(%)	75(%)	0.456
Caucasian	88(%)	100(%)	0.200
BMI	30.2 ± 1.5	26.4 ± 2.3	0.173
**Smoking status**			
Current smoker	42.4(%)	16.7(%)	0.101
Former smoker	39.4(%)	33.3(%)	0.456
Non-smoker	9.1(%)	41.6(%)	**0.018**
**Comorbidities**			
Diabetes mellitus	36.4(%)	58.3(%)	0.764
Hypertension	90.9(%)	91.7(%)	0.938
COPD	9.1(%)	16.7(%)	0.485
Hyperlipidemia	84.8(%)	66.7(%)	0.186
Chronic kidney disease	21.2(%)	58.3(%)	**0.017**
**Medications**			
ARB	6.1(%)	25(%)	0.077
ACEi	60.6(%)	50(%)	0.268
Statins	87.9(%)	91.7(%)	0.700
Nitrates	18.2(%)	33.3(%)	0.290
Beta-blockade	87.9(%)	83.3(%)	0.302
CCA	18.2(%)	8.3(%)	0.432
ASA	90.9(%)	91.7(%)	0.486
Other antiplatelet	(%)	(%)	
Insulin			
**Laboratory values**			
Total cholesterol	145.9±	164.3±	0.335
HDL	36.6±	35.11±	0.702
LDL	98.3±	84.4±	0.307
Triglycerides	165.2±	180.6±	1.000
Hg A1C	6.3±	6.3±	0.970
Serum creatinine	0.9±	1.3±	**0.001**

*Bold font indicates the significant data*.

## Discussion

This is the first study, to our knowledge, to describe venous calcification in terms of concurrent vein pathology, endothelial function, and patient risk factors. In this study, ~30% of the veins analyzed had calcium deposits. Veins with calcium had higher intimal thickness (Figure 2), increased extracellular collagen deposition (Figure 3), and decreased endothelial relaxation (Figure 4). Significant patient risk factors included age and preoperative SCr (Table 1).

Once believed to be a passive process of metabolic derangement, vascular calcification is now thought to result from dedifferentiation of VSMC to an osteoblast phenotype in response to injury. Osteoblasts responsible for bone calcification produce a primarily collagen-based ECM that is subsequently mineralized with hydroxyapatite, a calcium phosphate (16). Arterial calcification studies have demonstrated a similar pattern of collagen and calcium deposition (17). In the present study, we observed calcium deposits within areas of increased ECM collagen with more collagen per area in calcified veins. This suggests the presence of cell-mediated osteogenesis by osteoblast-type VSMC within veins as in arterial and bone calcium deposition.

The pathological deposition of vascular ECM facilitates not only mineralization but also the migration of VSMCs from media to intima, a key underlying process of IH (18). Chitalia et al. reported on the coexistence of IH and vascular calcification within brachial and radial arteries of CKD patients (19) while Janda et al. concluded that intimal thickness within the carotid artery predicted the presence and severity of calcification (20). Our study found a strong association (*r*^2^ = 0.184) between calcium and intimal thickness within the saphenous vein as well. This is of particular importance in assessing vein quality prior to arterial bypass as preexisting intimal thickness in veins predicts bypass graft failure (21).

The ideal venous bypass conduit has a functioning, intact endothelium, the primary regulator of vascular tone, and vessel wall hemostasis (22). The loss of endothelial function predisposes the vessel to vasospasm, platelet aggregation, and IH. In this study, we found that the presence of venous calcification predicted reduced endothelial-dependent relaxation.

The temporal organization of events cannot be concluded from these data. It is likely that an injurious venous environment related to cardiovascular risk factors causes loss of endothelial regulation and VSMC dedifferentiation. This may in turn lead to IH and calcium deposition. It is also possible that vein calcification preceded the development of IH as can be seen in postphlebitic vein segments or alternatively that endothelial dysfunction related to venous reflux was the primary event with subsequent development of the observed morphological changes. The precise sequence of events in a particular patient or group of patients may be heterogeneous. Sessa et al. demonstrated that saphenous vein endothelial function was reduced in diabetics, smokers, and patients with hypertension (23), while age, diabetes, and end-stage renal disease (ESRD) have long been associated with the development of arterial calcification (24). Not surprisingly, risk factors for saphenous vein calcification in our study were older age and elevated preoperative SCr. While smoking status, ESRD, and hypertension did not reveal a significant association with vein calcification, we suspect a larger sample size would ultimately reveal a trend mirroring arterial calcification risk factors.

There are several limitations with this study. The sample size was relatively small. Only acute variables were examined, and further studies with a larger sample size are needed to draw conclusions regarding venous calcification and long-term bypass patency rates. The innate variability between samples when using human tissue can be vast. The sections of vein analyzed were not implanted and, thus, may not represent the histology and function of grafted tissue. Further efforts using imaging modalities such as vein graft duplex US may allow for longitudinal assessment of calcification and assessment of its long-term effects on patency.

## Conclusion

The results of this study demonstrated that preexisting calcification in saphenous vein is a marker of poor conduit quality. HSVs with calcification can be characterized as having increased intimal thickness, increased ECM deposition, and reduced endothelial-dependent relaxation. Calcified saphenous vein is more common in older patients and those with reduced kidney function. Further studies are needed to determine if long-term graft patency rates are affected by preexisting vein calcification.

## Author Contributions

Conception and design: CG, KH, FL, JC-F, CB, RG, and PK. Acquisition of data: SP, CG, KH, AB, FL, and PK. Analysis and interpretation of data and review and final approval: SP, CG, KH, AB, FL, JC-F, RG, CB, and PK. Drafting of manuscript: SP, CG, and PK.

## Conflict of Interest Statement

The authors declare that the research was conducted in the absence of any commercial or financial relationships that could be construed as a potential conflict of interest.
